# Enhancing sensitivity of QCM for dengue type 1 virus detection using graphene-based polymer composites

**DOI:** 10.1007/s00216-021-03410-8

**Published:** 2021-06-06

**Authors:** Krongkaew Navakul, Chak Sangma, Pa-thai Yenchitsomanus, Suticha Chunta, Peter A. Lieberzeit

**Affiliations:** 1 Department of Chemistry, Faculty of Science, Kasetsart University, Chatuchak, Bangkok, 10900 Thailand; 2 Division of Molecular Genetics, Department of Research and Development, Faculty of Medicine Siriraj Hospital, Mahidol University, Bangkok, 10700 Thailand; 3 Department of Clinical Chemistry, Faculty of Medical Technology, Prince of Songkla University, Hat Yai, Songkhla, 90110 Thailand; 4 Department of Physical Chemistry, Faculty for Chemistry, University of Vienna, Währingerstraße 42, 1090 Vienna, Austria

**Keywords:** Molecularly imprinted polymers, Graphene oxide, Composites, Quartz crystal microbalance, Dengue type 1 virus

## Abstract

Graphene oxide-molecularly imprinted polymer composites (GO-MIP) have attracted significant attention as recognition materials in sensing due to their outstanding properties in terms of electrical and thermal conductivity, high mechanical modulus, and the comparably straightforward way to functionalize them. The aim of this study was to design a MIP-based sensor recognition material and enhance its sensitivity by blending it with GO for sensing a harmful dengue hemorrhagic fever pathogen, namely the dengue type 1 virus (DENV-1). Polymer composites comprising GO incorporated to an acrylamide (AAM)/methacrylic acid (MAA)/methyl methacrylate (MMA)/*N*-vinylpyrrolidone (VP) copolymer were synthesized and compared to the “pure” MIP, i.e., the copolymer without GO. The pure polymer revealed a zeta potential of + 9.9 ± 0.5 mV, whereas GO sheets prepared have a zeta potential of − 60.3 ± 2.7 mV. This results in an overall zeta potential of − 11.2 ± 0.2 mV of the composite. Such polymer composites seem appropriate to bind the positively charged DENV-1 particle (+ 42.2 ± 2.8 mV). GO-MIP coated onto 10-MHz quartz crystal microbalance (QCM) sensors indeed revealed two times sensitivity compared to sensors based on the pure MIP. Furthermore, GO-polymer composites revealed imprinting factors of up to 21, compared to 3 of the pure MIP. When plotting the sensor characteristic in a semilogarithmic way, the composite sensor shows the linear response to DENV-1 in the concentration range from 10^0^ to 10^3^ pfu mL^−1^. The corresponding limits of detection (S/*N* = 3) and quantification (S/*N* = 10) are 0.58 and 1.94 pfu mL^−1^, respectively. Furthermore, imprinted polymer composites selectively bind DENV-1 without significant interference: DENV-2, DENV-3, DENV-4, respectively, yield 13–16% of DENV-1 signal. The sensor requires only about 15–20 min to obtain a result.

**Graphical abstract:**

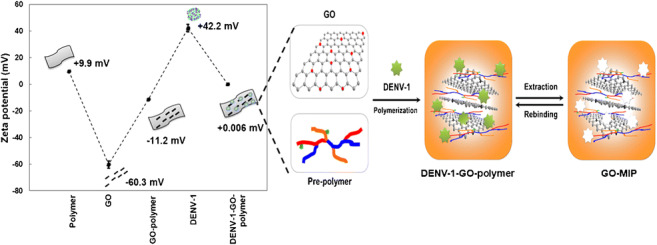

## Introduction

Dengue virus (DENV) is the cause of dengue hemorrhagic fever (DHF). It comprises four different serotypes, namely DENV-1, DENV-2, DENV-3, and DENV-4. DHF is a re-emerging public health problem for populations of tropical and sub-tropical regions, especially in the hyperepidemic region of Southeast Asia [1]. Although all four serotypes widely spread and circulate in most of this area, DENV-1 (36%) is the most common serotype in Thailand, followed by DENV-3 (27%), DENV-2 (23%), and DENV-4 (14%) [2]. A patient infected for the first time produces unique neutralizing antibodies against the respective particular serotype. Unfortunately, subsequent infection with another serotype may lead to antibody-dependent enhancement, producing non-neutralizing antibodies. Those promote virus entry into host cells, leading to more severe damage to internal organs [3]. Till now, no specific antiviral medication or vaccine exists for treating DHF and protecting patients against DENV, respectively [4]. Therefore, rapid detection and identification of DENV serotype infection is necessary for early diagnosis as well as for preventing severe damage and decreasing the risk of medical complications and death. Nowadays, rapid tests targeting dengue IgG/IgM and nonstructural proteins 1 (NS1) are utilized to detect DENV, but they offer only limited serotype classification [4]. For actually assessing the serotype, one therefore still needs genetic approaches, namely reverse transcriptase-polymerase chain reaction (RT-PCR). The method, however, does not allow for quantifying the virus load in the sample. For that purpose, one can use the standard plaque-forming unit (PFU) staining assay that requires cell cultivation [3]. Both methods are costly, laborious, and time-consuming. To tackle those issues, we herein present a rapid approach to both classify and directly quantify a DENV serotype, namely DENV-1 based on molecularly imprinted polymers (MIPs) and mass-sensitive detection. MIPs mimic the functions of natural receptors or antibodies by revealing selective properties via their shape, size, and chemical functionality [5]. MIP synthesis relies on the self-organization of functional monomers and cross-linkers around a template species. After polymerization and removing the template from the polymer, the matrix contains selective recognition sites complementary to the template. Those allow for selectively re-binding the respective templates, e.g., biomolecules [6–8]. Such biospecies are interesting target analytes for MIP-based sensing, because the polymers are usually much cheaper and more stable than recognition species from nature. Among various applications, surface MIPs for biospecies [9] have been synthesized successfully for wide a range of different viruses, such as the tobacco mosaic virus (TMV) [10] and influenza A subtypes (e.g., H5N1, H5N3, H1N1, H1N3, and H6N1) [11]. In addition, blending the polymer matrix with reinforcing materials, e.g., graphene oxide (GO), carbon nanotubes (CNTs), and metal nanoparticles, has led to high-performance composites. GO has received special attention in this regard, because among others, it is useful to enhance the thermal, mechanical, and electrical properties of the resulting composite [12]. Generally speaking, GO contains hydrophilic functional groups which can be functionalized in the polymer both covalently and non-covalently [13]. However, when preparing composites, one has to keep in mind that GO tends to aggregate and thus requires hydrophilic/hydrophobic groups present in the polymer to undergo strong polar-polar interactions [14]. They also impact on the system sometimes by their bulky size [15]. However, substantial research focusses on new strategies to modify surface-functionalized graphene oxide sheets for a range of applications [12, 14, 16].

For instance, Liu et al. published a polyethylene glycol functionalized nanographene oxide for delivery of water-insoluble cancer drug (SN-38) [17]. Cai *et al.* synthesized poly(ethylene amine) functionalized GO/silver nanocomposite to increase the stability and decrease the cytotoxicity of silver nanoparticles [18]. Chang et al. combined poly(methacrylamide) (PMAAM)-based MIP with GO to achieve a selective element to detect 2,4-dichlorophenol [19]. In this work, we assess the sensing properties of GO-MIP composite layers on quartz crystal microbalance (QCM) resonators to detect DENV-1. The DENV-1 serotype serves as a proof of principle, because it is widespread in Thailand.

## Materials and methods

### Synthesis of graphene oxide

GO synthesis followed Hummer’s method [20]. Briefly, we first dissolved graphite flakes (mesh size 300) by stirring them in a solution containing concentrated sulfuric acid and phosphoric acid in a ratio of 9:1 *(v/v)*, followed by slowly adding potassium permanganate. The mixture was then added to 150 mL of 3.33% (*v/v*) aqueous H_2_O_2_ solution, which led to a bright yellow color. The product was filtered via 0.2 μm Nylon membrane and washed with water before centrifugation at 5000 rpm for 30 min to obtain GO sheets.

### Preparation of dengue virus

The different dengue virus strains (DENV-1 to DENV-4) were cultured in C6/36 *Aedes albopictus* mosquito cells. All processes were carried out at biosafety laboratory level 2 under the standard safety protocol at the Department of Research and Development, Faculty of Medicine Siriraj Hospital, Mahidol University, as previously described [21]. The amount of each DENV serotype was assessed via a plaque-forming unit (PFU) staining assay using a monolayer of Vero cells. After incubating for 7 days, we stained the cell monolayer with 1% (*v/v*) crystal violet in 20% (*v/v*) ethanol to visualize circular plaques caused by DENV infection. The viruses were inactivated with β-propiolactone following a protocol of the Center for Disease Control and Prevention (CDC) before freezing them at − 70 °C.

### Fabrication of QCM transducers

Dual gold electrodes were generated on 10-MHz AT-cut quartz wafers (13.8 mm in diameter and 168 μm in thickness; Great Microtama Industries, Surabaya, Indonesia) using a brilliant gold paste (Heraeus; 12%) via screen printing [22]. Then, the quartz plates were baked in the oven at 400 °C for 4 h. Fundamental resonance and damping of QCM were monitored via an Agilent 8712ET network analyzer. QCM transducers with less than − 5 dB damping were chosen for further use.

### Preparation of DENV-1 imprinted graphene oxide-polymer composites

Previously published MIP for “negatively charged” influenza virus [11] (polymer A) served as the starting point for finding optimal MIP for DENV-1, because the envelopes of both viruses are made up of glycoproteins. However, both viruses present different surface charges. Therefore, polymer optimization included varying the amount of functional monomers, acrylamide (AAM), methacrylic acid (MAA), methyl methacrylate (MMA), and *N*-vinylpyrrolidone (VP) and keeping the amount of *N,N′*-(1,2-dihydroxyethylene) bisacrylamide (DHEBA) cross-linker and 2,2′-Azobis(2-methylpropionitrile) initiator constant at 47 and 1.5 mg, respectively, as shown in Table 1. This system had also proven useful as the matrix for electrochemical sensing of the dengue virus on electrodes enhanced with GO (i.e., without imprinting) [23]. Polymers A, B, and C were synthesized by dissolving all chemicals in 300 μL dimethyl sulfoxide (DMSO). The solution was pre-polymerized at 60 °C until just prior to reaching the gel point (approximately 30 min). In parallel, template virus stamps were prepared by coating 5 μL of DENV-1 standard corresponding to 1 × 10^5^ pfu mL^−1^ onto a bare glass plate, followed by sedimentation at 4 °C for 20 min.

**Table 1:** Composition of polymer recipes for optimizing MIP

Polymer types	Chemicals (mg)					
	Functional monomers				Cross-linker	Initiator
	AAM	MAA	MMA	VP	DHEBA	AIBN
A	15.3	11.0	6.5	6.7	47.0	1.5
B	5.0	12.0	12.0	6.7	47.0	1.5
C	5.0	20.0	0	6.7	47.0	1.5

**Scheme 1:**
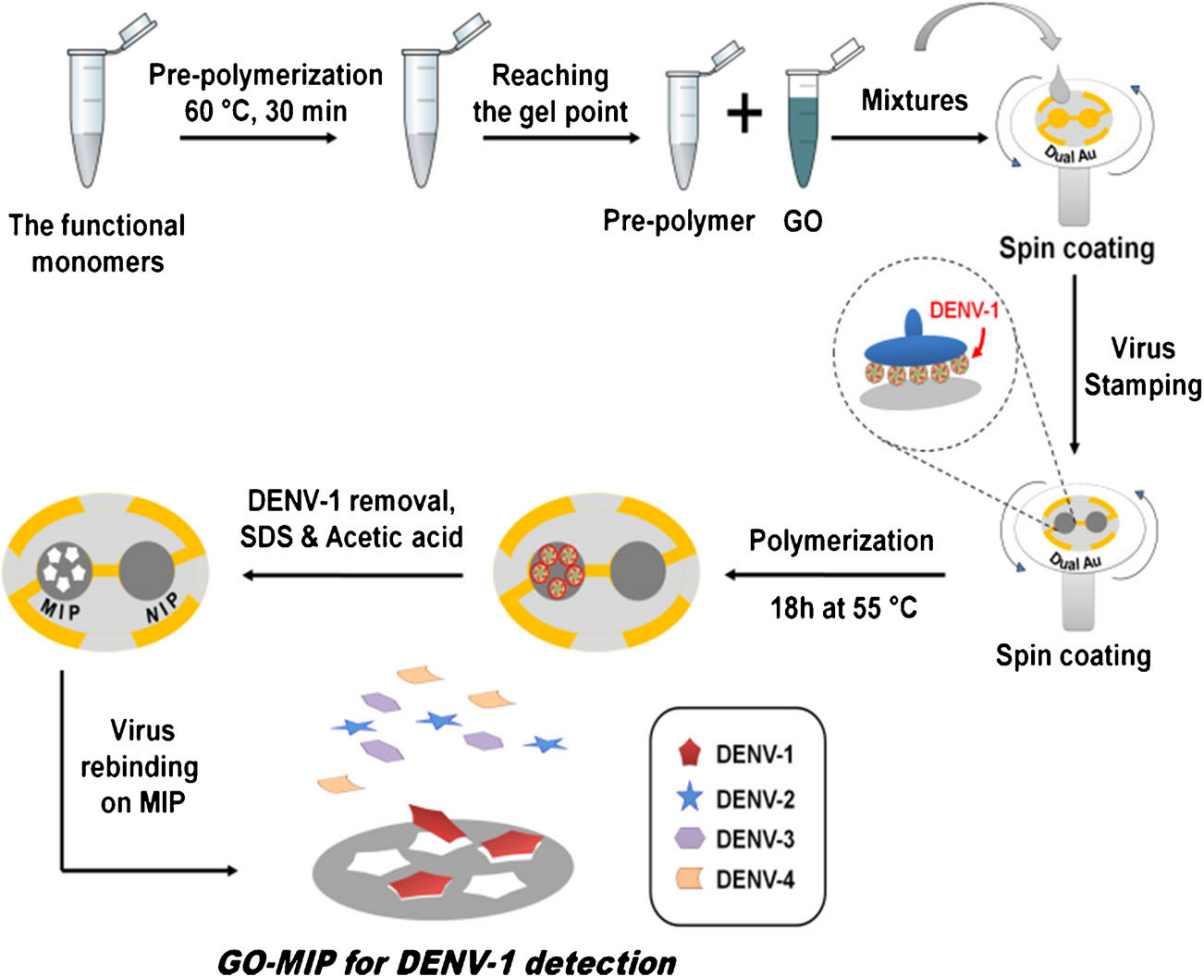
Schematic representation of GO-MIP and corresponding GO-NIP synthesis and their layer fabrication on QCM for detecting DENV-1

Scheme 1 represents the general approach of GO-polymer composite layer fabrication: First, a pre-polymer solution was prepared as previously mentioned. Then, we mixed a colloidal suspension of GO platelets in 0.01 M PBS, pH = 7.40 at c = 0.15 mg mL^−1^ to the desired pre-polymer matrix at a ratio of 3:2 (*v/v*). Those composite batches were spin-coated at 3000 rpm for 2 min over both QCM electrodes. Instantly, the virus template stamp was pressed into the pre-polymer composite film above one of the electrodes to yield the GO-MIP. The polymer on the untreated electrode leads to the so-called non-imprinted polymer (GO-NIP) for reference. Then, the polymer layer on QCM was completely polymerized at a 55 °C oven for 18 h. Finally, templates were removed by stirring in an aqueous solution containing 1 part 10% *(v/v)* acetic acid and 1 part 0.1% *(w/v)* sodium dodecyl sulfate (SDS) solution, respectively, followed by deionized water (DW) for 30 min each. In a similar way, we prepared sensors containing “standard” MIP and NIP (without GO).

### Characterization of polymer composites

Scanning electron microscopy (SEM) was carried out on a Quanta 450 FEI SEM with a voltage of 20.0 keV. All samples were coated with a thin layer of Au by sputter-coating prior to SEM examination. Atomic force microscopy (AFM) served for characterizing morphologies of polymer surfaces on QCM using a Bruker Instruments NanoScope 8 in contact mode with silicon nitride cantilevers at a 1 Hz scan rate. To measure the zeta potentials of all material interfaces in 0.01 M PBS, pH = 7.40 at 25 °C, we used a Malvern Zetasizer Nano-ZS90 instrument.

### QCM measurements

Dual-electrode QCM were placed into a custom-made measuring cell, which connected to a home-built oscillator circuit linked to a frequency counter (Agilent 53131A) for a read-out. A custom-made LabView routine transferred measurement data to a computer by a GPIB/USB interface. All experiments took place in a stop-flow mode at room temperature (25 °C) to minimize sample volumes. For sensing, we mounted each QCM in a custom-made measuring cell, followed by obtaining equilibrium baseline signal through adding 180 μL of 0.01 M PBS, pH 7.40. Then, the sensor was exposed to 180 μL of the respective DENV-1 standard in 0.01 M PBS until reaching equilibrium. This was followed by washing with a mixed solution containing 10% (*v/v*) acetic acid/0.1% (*w/v*) SDS in a ratio of 1:1, followed by DW to regenerate MIP.

## Results and discussion

### Optimizing polymer synthesis

**Fig. 1:**
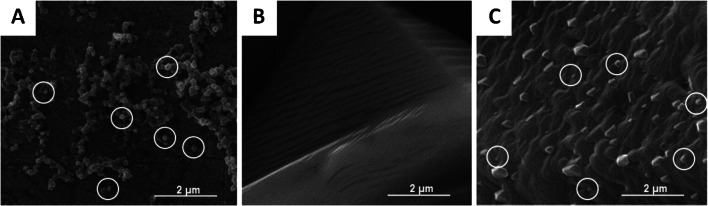
SEM images of DENV-1 in 0.01 M PBS, pH = 7.40 (**A**), GO in 0.01 M PBS, pH = 7.40 (**B**), and DENV-1 on GO (**C**). (DENV-1 particles are indicated in white circles)

**Fig. 2:**
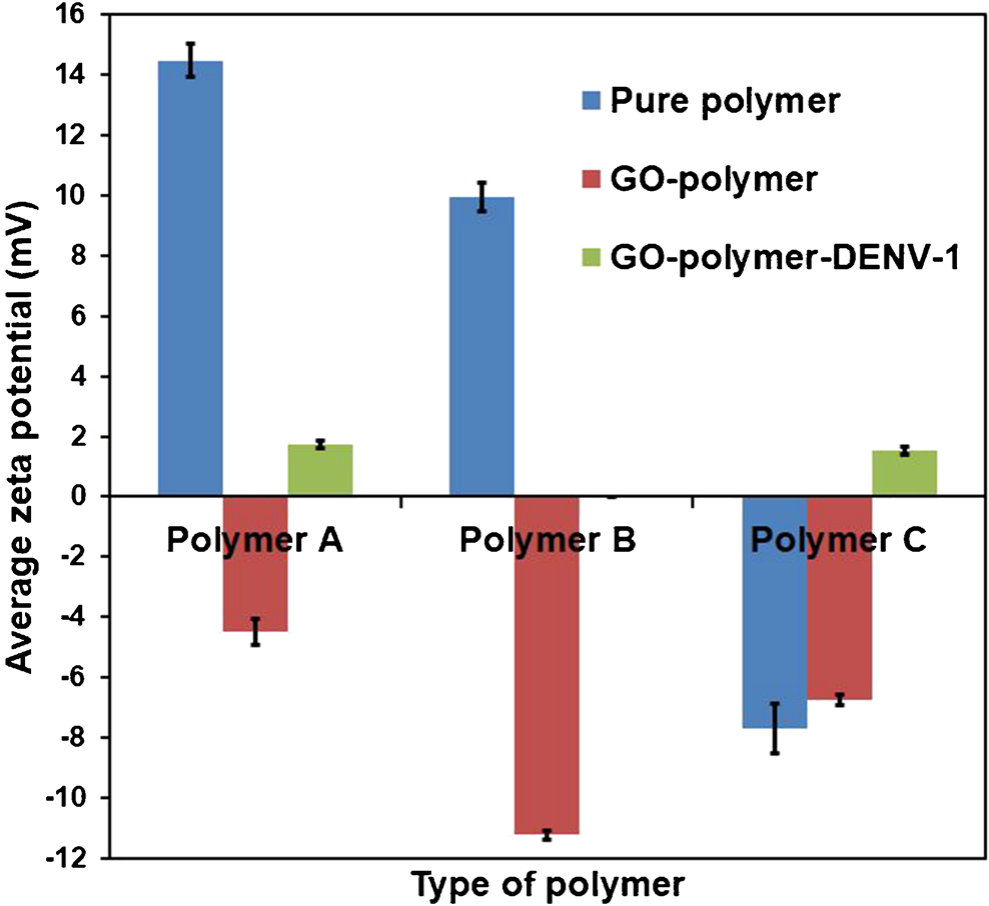
Zeta potentials of copolymers **A**–**C** (containing various ratios of AAM, MAA, MMA, VP), GO-polymer composites, and GO-polymer-DENV-1

GO and DENV-1 in 0.01 M PBS, pH = 7.40 have a net negative charge at − 60.3 ± 2.7 mV and a net positive charge at +42.2 ± 2.8 mV, respectively. Figure 1 collects SEM images of different surfaces, namely DENV-1 (Fig. 1A), GO (Fig. 1B), and GO with DENV-1 (Fig. 1C). Obviously, DENV-1 particles indeed show affinity toward the GO surface due to electrostatic interactions. Of course, those are inherently non-selective, but affine. Therefore, it seems reasonable to optimize affinity between the receptor—i.e., the MIP—and its target analyte beyond “just” imprinting. Especially in aqueous solutions, electrostatic interactions play an important role in that regard. Figure 2 summarizes the zeta potentials of copolymers A–C, and their composites with GO and GO/DENV-1, respectively. DENV-1 on the one hand has a positive surface charge totaling + 42.2 ± 2.8 mV and on the other hand comprises a hydrophilic moiety comprising both negatively and positively charged side chains in the same way as other biomolecules. These observations have led to choosing AAM, MAA, MMA, and VP, as functional monomers, because they can provide a polymer with compositions of positive and negative charges. However, polymers A and B also reveal a positive surface charge at the respective values of + 14.5 ± 0.5 mV and + 9.9 ± 0.5 mV, which makes them improbable candidates for binding a positively charged species such as DENV-1. In contrast to this, polymer C showed a negative surface charge at − 7.7 ± 0.8 mV due to the increasing amount of negatively charged MAA. However, that value still seems rather low. On the other hand, GO sheets after synthesis led to a zeta potential of − 60.3 ± 2.7 mV due to the large number of carboxyl functionalities present on the surface. They indeed lead to a more negative surface charge of the respective GO-polymer composites, namely − 4.5 ± 0.4 mV for polymer A, − 11.2 ± 0.2 mV for polymer B, and − 6.7 ± 0.2 for polymer C. After exposing all composites to a DENV-1 solution at a concentration of 10^3^ pfu mL^−1^, the zeta potentials of all polymers turned slightly positive again upon interaction: + 1.7 ± 0.1 mV for polymer A, + 0.006 ± 0.003 mV for polymer B, and + 1.5 ± 0.1 mV for polymer C. This indicates the inherent affinity of the GO-polymer to DENV-1, which is favorable for developing MIP. Overall, GO-polymer B reveals substantially higher negative surface potential than the other two composites which made it the best candidate for further experiments.

### Surface morphologies of classical polymers and composites

**Fig. 3:**
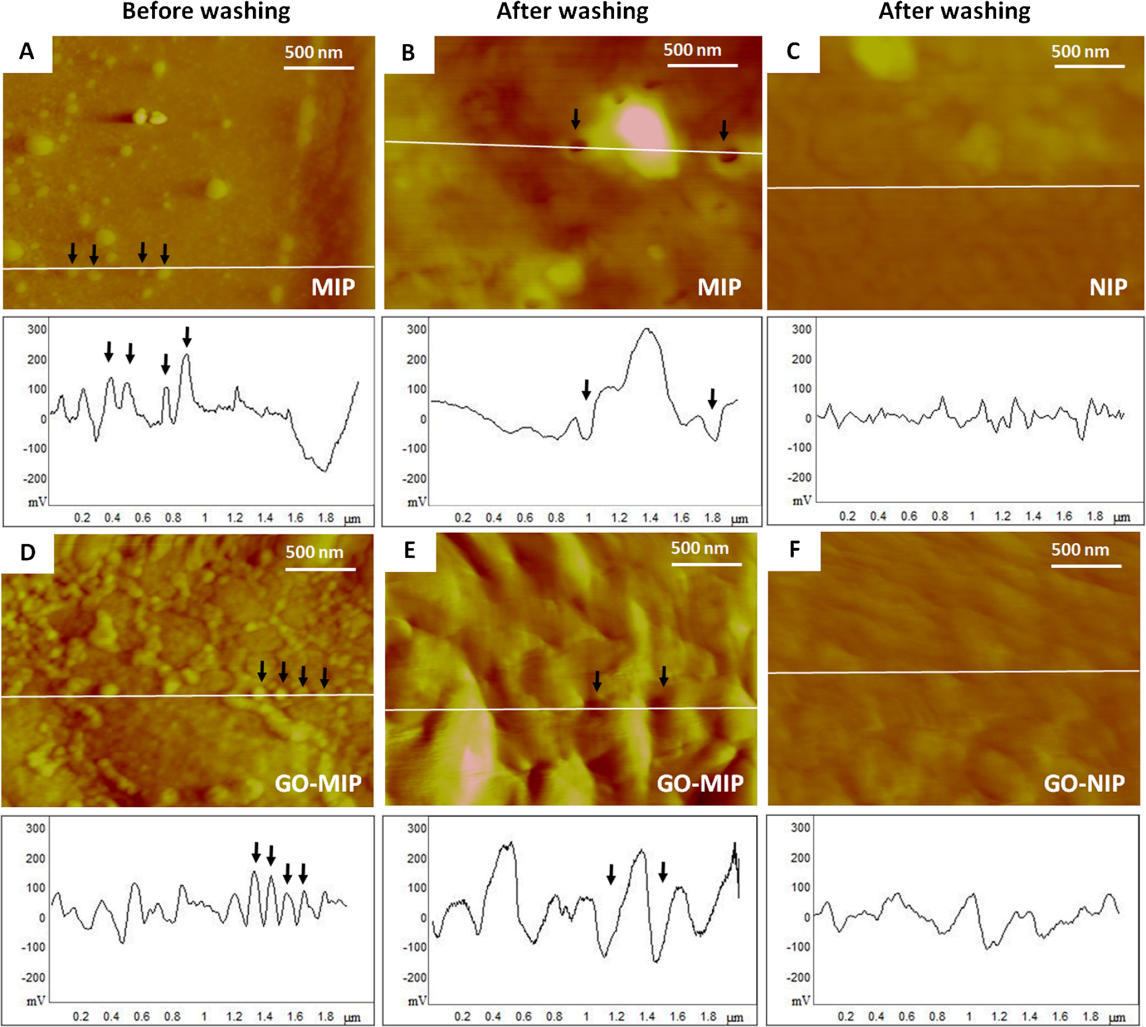
AFM images and corresponding surface roughness profiles of polymers and GO/polymer composites (black linear graphs represent the selected cross-sections of the corresponding AFM image to show surface roughness. Some bound virus particles and imprinted cavities are marked by dark arrows.): (**A**, **D**) MIP and GO-MIP before removing DENV-1 (**B**, **E**) MIP and GO-MIP after removing DENV-1 (**C**, **F**) NIP and GO-NIP

Figure 3 displays AFM images of different surfaces, namely DENV-1-MIP before (Fig. 3A) and after (Fig. 3B) washing out the template, and GO-MIP before (Fig. 3D) and after (Fig. 3E) the same steps, respectively, as well as their corresponding NIPs (Fig. 3C and F). Evidently, MIP surfaces before removing the virus are rough and reveal numerous independent roughly globular structures of DENV-1 with an average diameter of 96 ± 8 nm on the surface. Deviations from the ideal shape may result from AFM measurements: in the case that tips are not ideally rotationally symmetric, one can observe such issues. In the same way, one can clearly see virus particles on the surface of the GO-MIP composite (with an average diameter of 93 ± 7 nm). After removing the template, the MIP and GO-MIP surfaces also show similar roughly globular cavities with the respective diameters of 94 ± 5 nm and 88 ± 8 nm, corresponding to the size of DENV-1 particles. However, GO-MIP surfaces are rougher than those of MIP, because they contain GO sheets with sizes of 300–800 nm. Furthermore, neither NIP contains any of those features. These AFM images reveal that switching from the polymer to the composite does not change the shapes of the binding cavities revealed on the respective surface. They also clearly demonstrate that the three different polymer surfaces show drastically different morphologies.

### QCM sensing studies

**Fig. 4:**
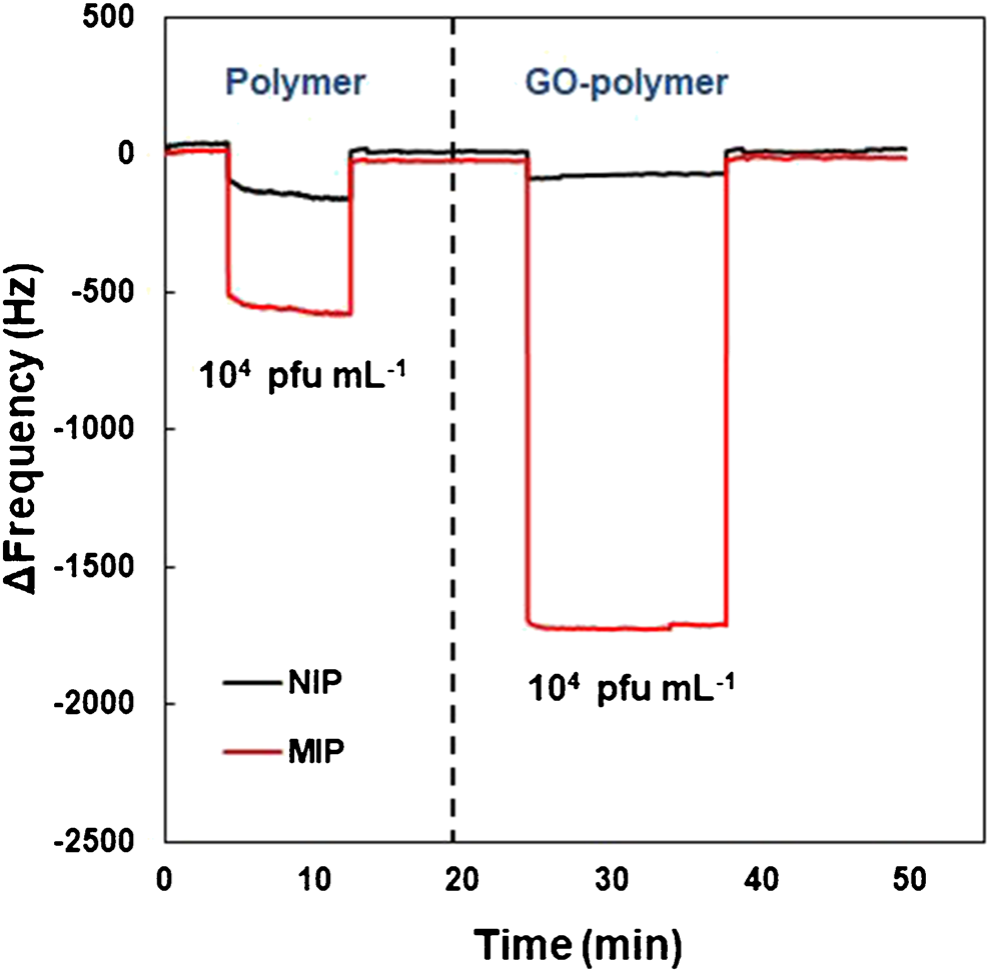
QCM frequency responses of DENV-1-MIP/NIP and GO-DENV-1-MIP/NIP toward a standard DENV-1 in 0.01 M PBS at c = 10^4^ pfu mL^−1^

Figure 4 shows two sets of dual-electrode QCM responses: the first is the result of exposing a device carrying a DENV-1-MIP and NIP, respectively, when exposing them to a 10^4^ pfu mL^−1^ DENV-1 standard solution. This leads to frequency shifts of − 595 Hz on the MIP side and − 197 Hz on the NIP side, corresponding to a – 399-Hz mass effect, which provides evidence for successful imprinting. However, the imprinting factor (i.e., the signal ratio MIP/NIP) is 3, which is rather low. The second sensor response is from a device coated with the two composites (GO-MIP and GO-NIP), respectively. In this case, exposing the device to the same standard solution of DENV-1 leads to signals of − 1690 Hz for GO-MIP and − 80 Hz for GO-NIP, hence a – 1610-Hz mass effect overall. The imprinting factor increases to 21, which has a large impact on sensitivity. One can trace back such different response behaviors to surface charges: both the polymer (+ 9.9 ± 0.5 mV) and DENV-1 (+ 42.2 ± 2.8 mV) have net positive surface charges, which obviously reduces the ability of the virus to efficiently bind to the MIP. The resulting sensor effect hence may be the result of comparably small enthalpic contributions combined with substantial entropy gain. Introducing GO into the polymer to yield GO-MIP, however, shifts the net surface charge of the thin film to negative values (− 11.2 ± 0.2 mV) as previously mentioned. Hence, GO substantially increases the affinity of the respective MIPs and results in improved sensor responses. Moreover, the composites respond to the analytes faster than the pure polymers: their negative charges also increase the probability that a virus particle actually reaches a binding site on the MIP surface as a result of electrostatic attraction. It also leads to the shorter time for the signal to reach equilibrium, namely from roughly 5 min to the range of 1 min. Both AFM images and QCM responses hence strongly support the claim of successful GO-MIP synthesis.

**Fig. 5:**
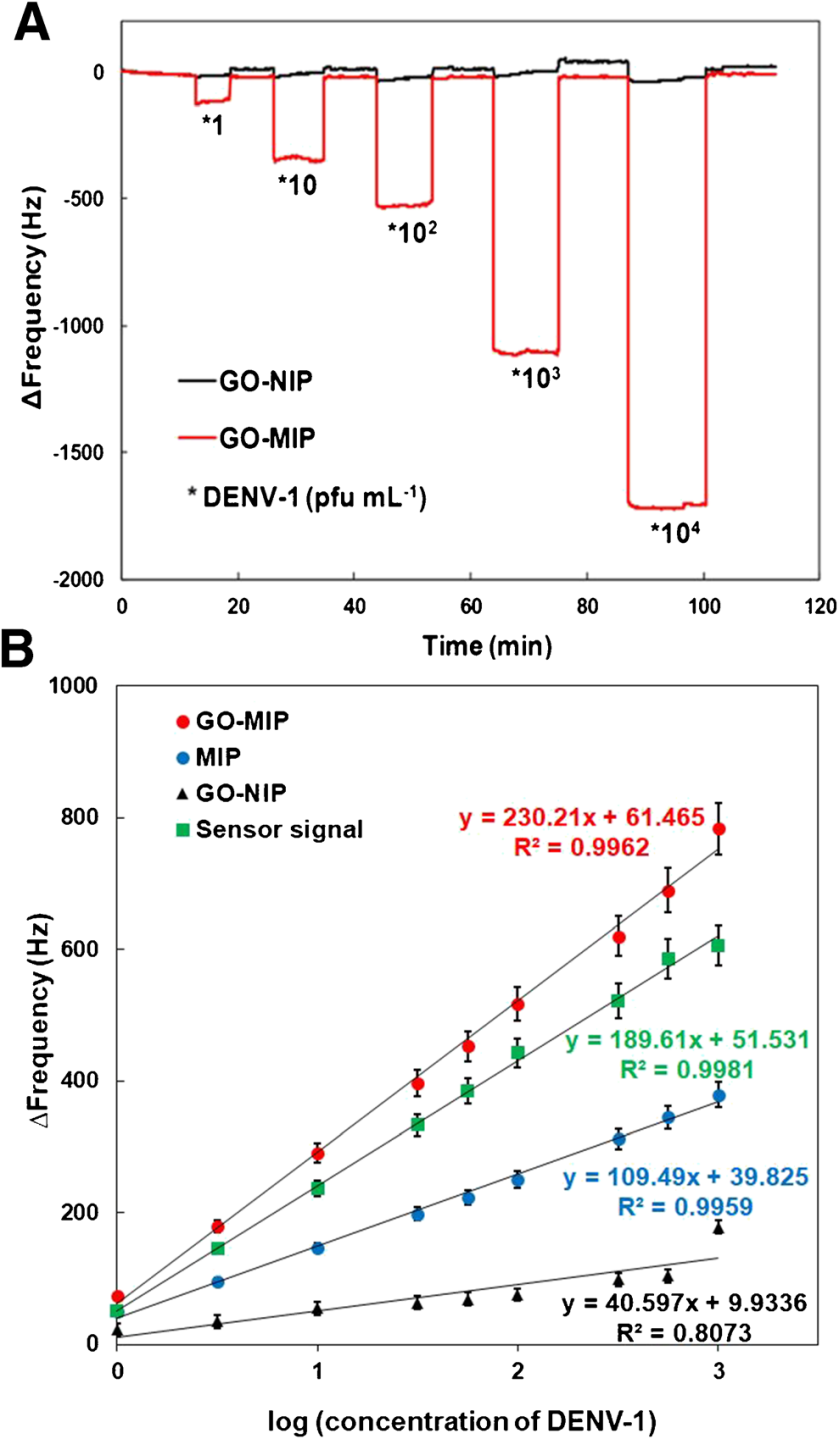
GO-MIP/NIP sensor responses toward standard DENV-1 in 0.01 M PBS with the range concentration of 10^0^ to 10^4^ pfu mL^−1^ (**A**); and linear response characteristic of GO-MIP, MIP, and GO-NIP toward standard DENV-1 in 0.01 M PBS with the range concentration of 10^0^ to 10^3^ pfu mL^−1^(**B**)

These promising results led us to investigate the sensor characteristics in a concentration range of 10^−1^ to 10^4^ pfu mL^−1^ DENV-1. Figure 5A shows the frequency responses of GO-MIP and GO-NIP, respectively, as a function of time. Obviously, frequency shifts increase with increasing DENV-1 concentrations with a dynamic range covering 10^0^ to 10^4^ pfu mL^−1^. At the lowest concentration (10^−1^ pfu mL^−1^), the sensor response was too small (Δf = − 26 Hz; data not shown) to be statistically significant at a noise level of roughly 10 Hz. Plotting the sensor characteristics in a semilogarithmic way reveals linear sensor characteristics of the GO-MIP sensor in the concentration range from 0 to 3 (i.e., 10^0^–10^3^ pfu mL^−1^), as one can see in Fig. 5B. Data fit is excellent with a correlation coefficient of R^2^ = 0.9981. Limits of detection (LOD) and quantification (LOQ) were calculated on the basis of a 10-Hz measurement noise, obtaining 0.58 and 1.94 pfu mL^−1^, respectively. Hence, the system is able to detect virus concentrations that correlate to early-stage infection with DENV-1. Compared to the MIP without graphene oxide, the composite is a factor of two more sensitive. Overall, the pure MIP led to LOD = 0.60 pfu mL^−1^ and LOQ = 2.0 pfu mL^−1^, which are higher than for the GO-MIP sensor. Therefore, rationally altering surface charge by synthesizing the composite pushes the sensitivity of the system toward the range required for actually applying those sensors in real-life settings, which is unusual for QCM-based sensing. However, imprinting also substantially contributes to sensitivity: the slope of the GO-NIP QCM sensor characteristic is (k = 40.60) is roughly 2.5 times lower than that of the MIP and almost six times lower, than for the GO-MIP, respectively. Compared to the standard plaque assay that utilizes cell cultivation for virus quantification, this sensor strongly reduces analysis time from at least a 7-day incubation for plaque assay to within 15–20 min for sensor measurement.

### Selectivity

**Fig. 6:**
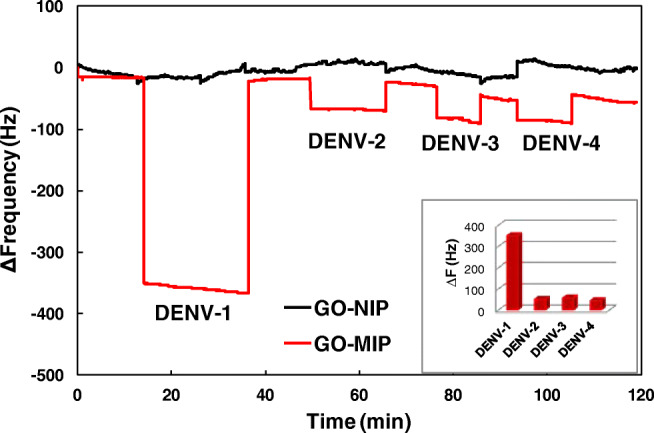
GO-MIP/NIP sensor responses toward each serotype of DENV at a concentration of 50 pfu mL^−1^. The inset shows the relative effect compared to the respective DENV-1 signal

As mentioned, there are four dengue virus serotypes (DENV-1 to DENV-4) that differ by the specific antigens on the virus surface. Therefore, it is imperative to characterize the selectivity of MIPs and composites. Figure 6 summarizes the sensor responses of DENV1-GO-MIP and NIP, respectively, toward each serotype of DENV at a concentration of 50 pfu mL^−1^. The sensor responses for DENV-1, 2, 3, and 4, are − 350, − 51, − 57, and − 44 Hz, respectively, corresponding to the relative effect of 100%, 15%, 16%, and 13% of the DENV-1 signal. Such comparably low cross-reactivity between serotypes is even more surprising considering that they are 65–70% homologous among each other [24]. Thus, the selectivity of MIP composites fits well with the functional differences between serotypes.

## Conclusions

Graphene-based polymer composites allow for rationally altering the interaction properties of sensor layers by influencing surface charge. Though seemingly a “simple” step in sensor development, it shows substantial effects for detecting DENV-1 virions: GO-MIP composites lead to realistic limits of detection on QCM by increasing sensitivity by a factor of two compared to unmodified MIP. This study model of DENV-1 is promising for further studies on sensor arrays quantifying and identifying specific DENV serotypes in one measuring step of roughly 20 min. This is much faster than any method based on cell culture or on nucleic acid amplification. In principle, the approach is expected to be feasible for mass-production, when using—harmless—pseudoviral particles for templating, i.e., virions lacking the viral genome.
